# Supplementary material for: PKA at a Cross-Road of Signaling Pathways Involved in the Regulation of Glioblastoma Migration and Invasion by the Neuropeptides VIP and PACAP
Source: Cancers (Basel). 2019 Jan 21;11(1):123. doi: 10.3390/cancers11010123 (PMC6356933; doi:10.3390/cancers11010123)
Supplement: Supplementary file 1 [file cancers-11-00123-s001.pdf]

Supplementary Materials: PKA at a Cross-Road of Signaling Pathways Involved in the Regulation of Glioblastoma Migration and Invasion by the Neuropeptides VIP and PACAP

Souheyla Bensalma, Soumaya Turpault, Annie-Claire Balandre, Madryssa De Boisvilliers, Afsaneh Gaillard, Corinne Chadéneau and Jean-Marc Muller

Table S1. Expression of mRNAs encoding the components of the VIP-receptor system in C6 and U87 glioblastoma cell lines.

Cell Lines	VIP	PACAP	VPAC1	VPAC2	PAC-1
C6	$2.6 \times 10^{-5} \pm 0.1 \times 10^{-5}$	$2.25 \times 10^{-7} \pm 0.6 \times 10^{-7}$	$8.2 \times 10^{-7} \pm 0.1 \times 10^{-7}$	$1.1 \times 10^{-5} \pm 0.12 \times 10^{-5}$	$5.25 \times 10^{-7} \pm 0.4 \times 10^{-7}$
U87	$1 \times 10^{-5} \pm 0.4 \times 10^{-5}$	-	4×10^{-7}	-	$3 \times 10^{-5} \pm 0.33 \times 10^{-5}$

Real-time RT-PCR analysis of mRNAs encoding VIP, PACAP, their receptors (VPAC1, VPAC2 and PAC1) and GAPDH (used as an internal control) in C6 and U87 cells. Expression was normalized to GAPDH mRNA expression. The primer sequences utilized in these experiments are presented in Table S2. Analysis is performed according to the ΔCt method. Data are the mean ± SD of three independent experiments, each performed in triplicate.

Table S2. primer sequences used for RT-qPCR analysis of expression of mRNAs encoding the components of the VIP-receptor system in C6 and U87 glioblastoma cell lines.

Target	Direction	Nucleotide Sequence (5′-3′)
PACAP (human)	Forward	TGCTGTCCATGCTTCTCATC
	Reverse	CAGTGCAGGAGGGTAGGAAG
VIP (human)	Forward	CCGCCTTAGAAAACAAATGG
	Reverse	TTGTCATCAGCTTTGCTCCA
VPAC1 (human)	Forward	ACAAGGCAGCGAGTTTGGAT
	Reverse	GTGCAGTGGAGCTTCCTGAAC
VPAC2 (human)	Forward	CGTGAACAGCATTCACCCAGAAT
	Reverse	CGTGACGGTCTCTCCCACAT
PAC1 (human)	Forward	GTGCAGTGGAGCTTCCTGAAC
	Reverse	ACAAGGCAGCGAGTTTGGAT
GAPDH (human)	Forward	TGCTGTCCATGCTTCTCATC
	Reverse	GACAAGCTTCCCGTTCTCAG
PACAP (rat)	Forward	ATGTCGCCCACGAAATCCTT
	Reverse	TGTCTGTGAAGATGCCGTCC
VIP (rat)	Forward	GGAAAGACCCAAGGAGGCAC
	Reverse	CACTGAAGAAGGTGGCCCAT
VPAC1 (rat)	Forward	GCTCATCCCCCTGTTTGGAA
	Reverse	ACGGATGCTGGGATTTGGAG
VPAC2 (rat)	Forward	TTCCTCCCAGCAGGTGTTTC
	Reverse	GTCCCAGCAACCTGTGTCTT
PAC1 (rat)	Forward	CTCTCCCTGACTGCTCTCCT
	Reverse	CCCCCATGTCTGTGATCTCC
GAPDH (rat)	Forward	GGTCTACATGTTCCAGTATGAC
	Reverse	GTTGATGACCAGCTTCCCATTCT

© 2019 by the authors. Licensee MDPI, Basel, Switzerland. This article is an open access article distributed under the terms and conditions of the Creative Commons Attribution (CC BY) license (http://creativecommons.org/licenses/by/4.0/).
